# Sports gambling in the Americas: the rise of invisible risks

**DOI:** 10.1016/j.lana.2025.101211

**Published:** 2025-08-08

**Authors:** The Lancet Regional Health – Americas

Online sports betting has been growing rapidly across the Americas, driven by gamified apps, aggressive advertising and sponsorship initiatives, and a lack of effective regulation in many countries. The 2026 FIFA Men's World Cup, which will be held in the USA, Canada, and Mexico, represents a critical moment of mass exposure to betting advertisements. With a year until the tournament, there is time to assess the health, economic, and social impacts of online sports gambling.

A Systematic Review published in 2024 found that approximately 61.3% of adults in North America reported gambling within the past 12 months, with 13.8% of those engaging in risk gambling activities. This includes individuals who meet the criteria for problematic gambling or gambling disorder, a mental health condition recognized in both the DSM-5 and ICD-11, defined by a persistent pattern of gambling despite significant distress or impairment in daily functioning. Among adolescents, 33.7% reported gambling, with 27.8% of those engaging in risk gambling activities. A Systematic Review from Latin America and the Caribbean, mainly from Brazil, Ecuador, Mexico, and Peru, reported a variable prevalence of gambling disorder, ranging from 1.1% to 38.2%, with Brazil showing the highest rates (20.4–38.2%). Gambling disorder is more common among people in deprived areas, those who do not have a high school diploma or equivalent, and those with low income or unemployment.

Gambling, which includes activities such as casino games, lotteries, and online sport betting, poses significant risks to mental health. Previous publications have shown close associations with anxiety, depression, and obsessive-compulsive disorder. Moreover, gambling and gambling disorder significantly increase the risk of suicidality and self-harm. Wardle and colleagues reported that young adults aged 16–24 years who experience an increase in gambling severity over time are at higher risk of suicide attempts. Beyond mental health, gambling can lead to severe financial and social consequences, including debt, family disruption, and domestic violence, affecting not only individuals but also their families and communities.

Since the legalization of sports betting in the USA (2018) and Canada (2021), major sports leagues have formed partnerships with gambling brands, signalling a shift away from their traditional opposition and raising concerns about the normalisation of gambling within sports culture. Athletes have also increased their exposure to gambling through sponsorships and advertising. In Brazil, online sports betting was officially legalised in 2023; however, the regulatory landscape varies. Argentina regulates sports betting at the provincial level. In Chile, regulation is still under debate. Oversight varies by country; some, like the USA, leave decisions to states, a few of which (such as Utah and Hawaii) still ban all forms of sports betting. Effective regulation is needed to protect public health, prevent unlawful activities, and ensure fair play.

The USA online sports-betting market is projected to reach about US$25 billion in annual revenue by 2028, and the total Latin American online gambling market is expected to reach $12 billion in 2028, led by Brazil, Mexico, and Colombia. In May 2025, a major Brazilian food retail chain reported that online sports betting has significantly impacted consumers' purchasing power, with betting now making up nearly a third of the country's food sector turnover. This trend might reduce the amount of money households can allocate to food, potentially worsening food insecurity for many families. In vulnerable economies, online betting is often promoted as a quick solution to financial hardship, especially where inflation, informal work sectors, and unemployment are high, creating a cycle of financial insecurity and repeated losses.

While we celebrate the importance of sports for health, human development, and social integration, events like the FIFA Men's World Cup also create major opportunities for the betting industry through increased advertising and sponsorship, which help normalize and raise the visibility of betting. This is similar to how the tobacco industry and alcohol industries have used sports sponsorship to promote their products and influence public perception. Unlike with tobacco, we already have strong evidence of gambling's negative health and wellbeing effects, giving us an opportunity to act early.

As online sports betting continues to expand across the Americas, *The Lancet Regional Health—Americas* is committed to advancing dialogue and action on this pressing public health issue. We encourage policymakers, researchers, and community leaders to prioritise effective and independent regulation and to establish clear limits on marketing and algorithmic targeting. Cross-border educational efforts and international collaboration are essential to address this complex challenge.

